# Development and validation of a gene expression-based nomogram to predict the prognosis of patients with cholangiocarcinoma

**DOI:** 10.1007/s00432-023-04858-0

**Published:** 2023-05-24

**Authors:** Wei Wang, Chen Wu, Lijun Xu, Peilin Li, Kai Wang, Guangbing Li, Shanshan Zhao, Yongsheng Li, Xiaoyu Fan, Weifeng Wang, Meizhen Hu, Jing Wu, Shifeng Xu

**Affiliations:** 1 Interventional Department, The Second Hospital of Shandong University, Jinan, 250033 China; 2 Department of Hepatobiliary Surgery, Shandong Provincial Hospital, Shandong First Medical University, Jinan, 250021 China; 3 Department III of Radiotherapy, The Second People’s Hospital of Dezhou City, Dezhou, Shandong China; 4 Department of Hepatobiliary Surgery, The Second People’s Hospital of Dezhou City, Dezhou, Shandong China; 5 Shanghai OrigiMed Co., Ltd, Shanghai, China; 6 Mianyang Lide Electronics Co., LTD, Mianyang, China

**Keywords:** Cholangiocarcinoma, Gene mutation, Clinicopathological information, Nomogram, Prognosis

## Abstract

**Aim:**

To establish and validate a prognostic nomogram of cholangiocarcinoma (CCA) using independent clinicopathological and genetic mutation factors.

**Methods:**

213 patients with CCA (training cohort *n* = 151, validation cohort *n* = 62) diagnosed from 2012 to 2018 were included from multi-centers. Deep sequencing targeting 450 cancer genes was performed. Independent prognostic factors were selected by univariate and multivariate Cox analyses. The clinicopathological factors combined with (A)/without (B) the gene risk were used to establish nomograms for predicting overall survival (OS). The discriminative ability and calibration of the nomograms were assessed using C-index values, integrated discrimination improvement (IDI), decision curve analysis (DCA), and calibration plots.

**Results:**

The clinical baseline information and gene mutations in the training and validation cohorts were similar. SMAD4, BRCA2, KRAS, NF1, and TERT were found to be related with CCA prognosis. Patients were divided into low-, median-, and high-risk groups according to the gene mutation, the OS of which was 42.7 ± 2.7 ms (95% CI 37.5–48.0), 27.5 ± 2.1 ms (95% CI 23.3–31.7), and 19.8 ± 4.0 ms (95% CI 11.8–27.8) (*p* < 0.001), respectively. The systemic chemotherapy improved the OS in high and median risk groups, but not in the low-risk group. The C-indexes of the nomogram A and B were 0.779 (95% CI 0.693–0.865) and 0.725 (95% CI 0.619–0.831), *p* < 0.01, respectively. The IDI was 0.079. The DCA showed a good performance and the prognostic accuracy was validated in the external cohort.

**Conclusion:**

Gene risk has the potential to guide treatment decision for patients at different risks. The nomogram combined with gene risk showed a better accuracy in predicting OS of CCA than not.

**Supplementary Information:**

The online version contains supplementary material available at 10.1007/s00432-023-04858-0.

## Introduction

Cholangiocarcinoma is an epithelial cell malignancy located within the biliary tree united by late diagnosis and poor outcomes (Razumilava and Gores 2014). The incidence of CCA has increased globally over the past few decades. According to their anatomical location, CCAs are classified into intrahepatic (iCCA), perihilar (pCCA), and distal (dCCA) (Rizvi et al. 2018). The tumor-node-metastasis (TNM) staging system is critical for prognostic prediction and risk stratification (Gaag et al. 2012). Although resection and curative liver transplantation are options for selected patients with pCCA, 5-year survival rates are very low, ranged 25–40% (Mazzaferro et al. 2020). The established standard of care includes first-line (gemcitabine and cisplatin), second-line (FOLFOX), and adjuvant (capecitabine) systemic chemotherapy (Kelley et al. 2020). The clinicopathological information and treatment choice were all influencing the prognosis of CCA. Hence, the current anatomical-based staging system is not sufficient to predict the prognosis of CCA.

Prognosis differences might be related to biological heterogeneity, and molecular investigation might reveal biomarkers that can be used to predict prognosis and guide treatment for patients in different risk groups. With the improvement of detection technology, many gene mutations were found to be related to the prognosis of CCA, such as FGFR2 (Makawita et al. 2020), HAMP (Wang and Du 2021), GLUT-1 (Labib et al. 2019), KRAS (Dong et al. 2022), and so on. Many gene mutation-based target tyrosine kinase inhibitors were attempted to improve the prognosis of CCA (Andersen et al. 2012). But in fact, to guide individual treatment for patients, new biomarkers that reflect tumor heterogeneity are still needed. Meanwhile, a new prognosis evaluation system including gene mutation information should be established for CCA.

In this study, 450 cancer-related gene expression was sequenced and analysis aimed to identify and validate a gene expression signature that predicts OS in patients with CCA. Moreover, we combined genomic and clinical variables to generate a nomogram model with better predictive power than clinical risk factors for OS. An external cohort showed similar results. The discriminative ability and calibration of the nomogram with or without genomic information were evaluated using C-index values, integrated discrimination improvement (IDI), calibration plots, and decision curve analysis (DCA). Overall, we highlight the genomic information in CCA prognosis prediction and treatment selection.

## Methods

### Clinical specimens and study design

Totally 213 CCA patients were admitted in this study from the Second Hospital of Shandong University and the Shandong Provincial Hospital, which were divided into training cohort (*n* = 151) and validation cohort (*n* = 62) according to the admitted sequence. No patient had received any anti-tumor therapy before the pathological confirmation except directly resection. On accounting of quite different prognosis of different position of CCAs, and mixed hepatocellular cholangiocarcinomas have emerged as a distinct subtype of primary liver cancer (Razumilava and Gores 2014), we excluded the iCCAs in our study to eliminate the influence of iCCAs arising in cirrhosis. The inclusion criteria included pathologically confirmed cholangiocarcinoma; ECOG ≤ 2; clinicopathological, gene mutation and the following-up information were completed. The exclusion criteria included complicated with other tumors; not confirmed by pathology; did not receive any antitumor therapy; loss to follow-up.

All patients underwent staging contrast-enhanced CT scans or MRI, if necessary 18F-fluorodeoxyglucose positron emission tomography (18FDG-PET) also was recommended. Two radiologists independently reassessed all imaging scans, and the third radiologist was involved to resolve any disagreements. We restaged all patients according to the 8th edition of the American Joint Committee on Cancer Staging Manual. Surgery was preferred if available. The systemic treatment including adjuvant chemical therapy after resection; chemical therapy alone if the tumor was un-resectable, and the first line was gemcitabine and cisplatin, followed by FOLFOX if failed; target tyrosine kinase inhibitors were attempted if gene mutation recommended; immune checkpoint inhibitors were not used commonly mostly due to non-medical insurance and low willingness-to-pay. This retrospective analysis of anonymous data was approved by the institutional ethics review boards of the Shandong Provincial Hospital (ethics approval number: LCYJ: NO. 2019-081), and informed consent was waived by the ethics review boards.

### Procedures

We extracted at least 50 ng of DNA from each 40mm^3^ formalin-fixed, paraffin-embedded tumor sample using the QIAamp DNA FFPE Tissue Kit according to the manufacturer's protocol. The hybridization capture panel captured all coding exons of 450 tumor-related genes and selected introns of 39 commonly rearranged genes. Illumina NextSeq-500 was used to capture and sequence genes from FFPE samples and matched paracancerous samples. Sequencing results were further analyzed for single nucleotide variations (SNV), long- and short-range insertions and deletions (Indels), copy number variations (CNVs) and gene rearrangement/fusion structural variants. OrigiMed, an accredited and CLIA-certified College of American Pathologists laboratory, performed the genomic profiling using the YuanSu 450 panel (Cao et al. 2019).

### Gene mutation and critical clinicopathological information

The main endpoint was the overall survival (OS). We calculated OS to death from any reason. In the training cohort, expression profiling study was performed and which the mutation frequency was higher than 5% was selected to be the potential prognostic candidates. If gene candidates were correlated with the OS were analyzed, using univariate and multivariate Cox analysis, in which both were positive confirmed to be a prognostic gene mutation in this study. A novel gene mutation score was established by the number of the prognostic gene mutations, and all the patients were divided into three risk of groups: the low-risk (gene mutation score = 0), median-risk (gene mutation score = 1) and high-risk (gene mutation score ≥ 2) group. The overall survival curve of patients in different groups was compared.

Albumin–bilirubin (ALBI) grade was a novel independent prognostic factor of CCA (Wang et al. 2018). The ALBI score is 0.66 × log_10_TBIL (μmol/l)− 0.085 × ALB (g/l). To increase the degree of discrimination, we modified the ALBI grade named mALBI. The mALBI grade was defined by the resulting score (grade 1: ≤  − 2.60; grade 2: − 1.39 to − 2.27; grade 3:− 2.27 to − 2.60; grade 4: >  − 1.39) (Tokunaga et al. 2021).

Ca19-9 was a classical tumor biomarker of CCA (Wannhoff and Gotthardt 2019). Both the Ca19-9 level before and after resection were reported to be related to the prognosis of CCA (Jiang et al. 2021). So, we established a new tumor biomarker named Ca19-9 score, which was cumulated according the Ca19-9 before and after the resection if higher than 1000 u/ml.

### Statistical analysis

Comparisons between the training and test cohort were performed using the *χ*^2^ test when appropriate. OS was calculated using the Kaplan–Meier method and hazard ratios (HRs) were calculated using a univariate Cox regression analysis. The gene mutation score as a predictor for systemic treatment efficacy was analyzed in the training cohort and validated in the external test cohort. Univariate and multivariate Cox regression analyses were performed to select the independent prognostic factors including the gene mutation score and all clinicopathological information. The *p* value threshold was 0.05 (*p* > 0.05) for removing non-significant variables from the analysis. Correspondingly, significant variables (*p* ≤ 0.05) remained in the final Cox model. Covariates included the gene mutation score (0, 1, ≥ 2), mALBI (1–4), Ca19-9 score (0–2), stage, systemic treatment (no, unregular, and regular systemic treatment) and surgery (radical operation or not).

The rms package in R was used to formulate nomograms. For the generation of nomograms, we used the coefficients from a multivariable Cox regression model. The discriminative ability and calibration of the nomograms with or without the gene mutation score were assessed using C-index values, integrated discrimination improvement (IDI), calibration plots, and decision curve analysis (DCA). R (version 3.2.1) and SPSS (22.0) were used for the statistical analyses. Statistical tests were conducted two-sidedly, and *p* values less than 0.05 were considered significant.

## Results

### Baseline clinical data of the subjects

In this retrospective study, 151 CCA patients were included in the training cohort and 62 patients in the validation cohort. The baseline clinical data, including but not limited age, sex, tumor stage, radical surgery or not, mALBI, Ca19-9 score, systemic treatment or not, were compared between the two groups, and no significantly difference was found. Detailed information was shown in Table 1.

**Table 1:** The Clinical characteristics of patients in the training and validation cohorts

	The training cohort (151%)	The validation cohort (62%)	*Χ*^*2*^	*p* value
Sex ratio (*F*%)				
Male	111 (73.5)	41 (66.1)	1.172	0.5566
Female	40 (26.5)	21 (33.9)		
Age (y)				
< 60	66	23 (37.1)	0.790	0.6737
≥ 60	85	39 (62.9)		
Tumor stage				
I	36 (23.8)	13 (20.9)	2.354	0.5023
II	89 (58.9)	33 (53.2)		
III and IV	25 (16.5)	6 (9.7)		
Radical surgery				
Yes	119	46	0.536	0.764
No	32	16		
Systemic treatment				
Regular	77 (50.9)	43 (69.3)	6.243	0.099
Not regular	48 (31.8)	11 (17.7)		
No	26 (17.2)	8 (12.9)		

### The gene mutation was similar in the training and validation cohort

**Fig. 1:**
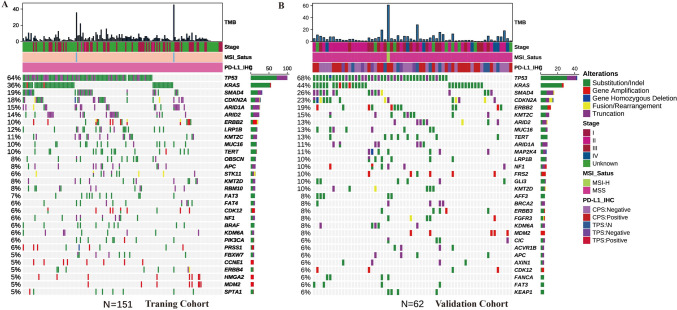
The gene mutation information in the training and validation cohort

The TOP10 gene mutations in training cohort were TP53(64%), KRAS (36%), SMAD4(19%), CDKN2A (18%), ARID1A (15%), ARID2(14%), ERBB2(10%), LRP1B (12%), KMT2C (11%), and MUC16(10%); and TP53(68%), KRAS (44%), SMAD4(26%), CDKN2A (23%), ERBB2(19%), KMT2C (15%), ARID2(13%), MUC16(13%), TERT(13%), and ARID1A(11%) in the validation cohort. The common mutation included substitution/Indel, gene amplification, gene homozygous deletion, fusion/rearrangement, and truncation. Detailed information is shown in Fig. 1.

### The gene mutation was correlated with the prognosis of CCA

**Fig. 2:**
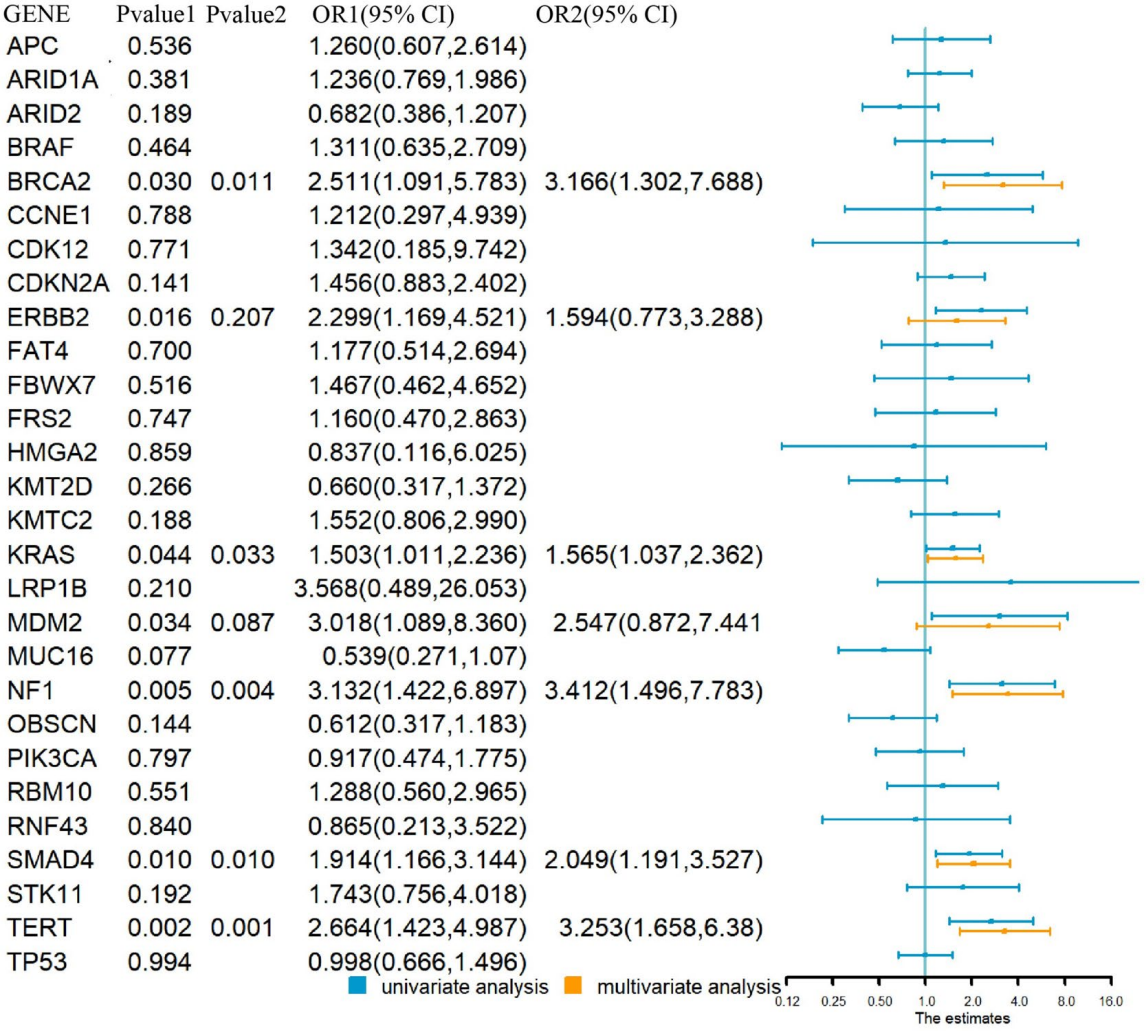
The relationship of gene mutations and overall survival of CCA. Note: Pvalue1: univariable Cox analysis; Pvalue2: multivariable Cox analysis. *OR* Odds ratio

**Fig. 3:**
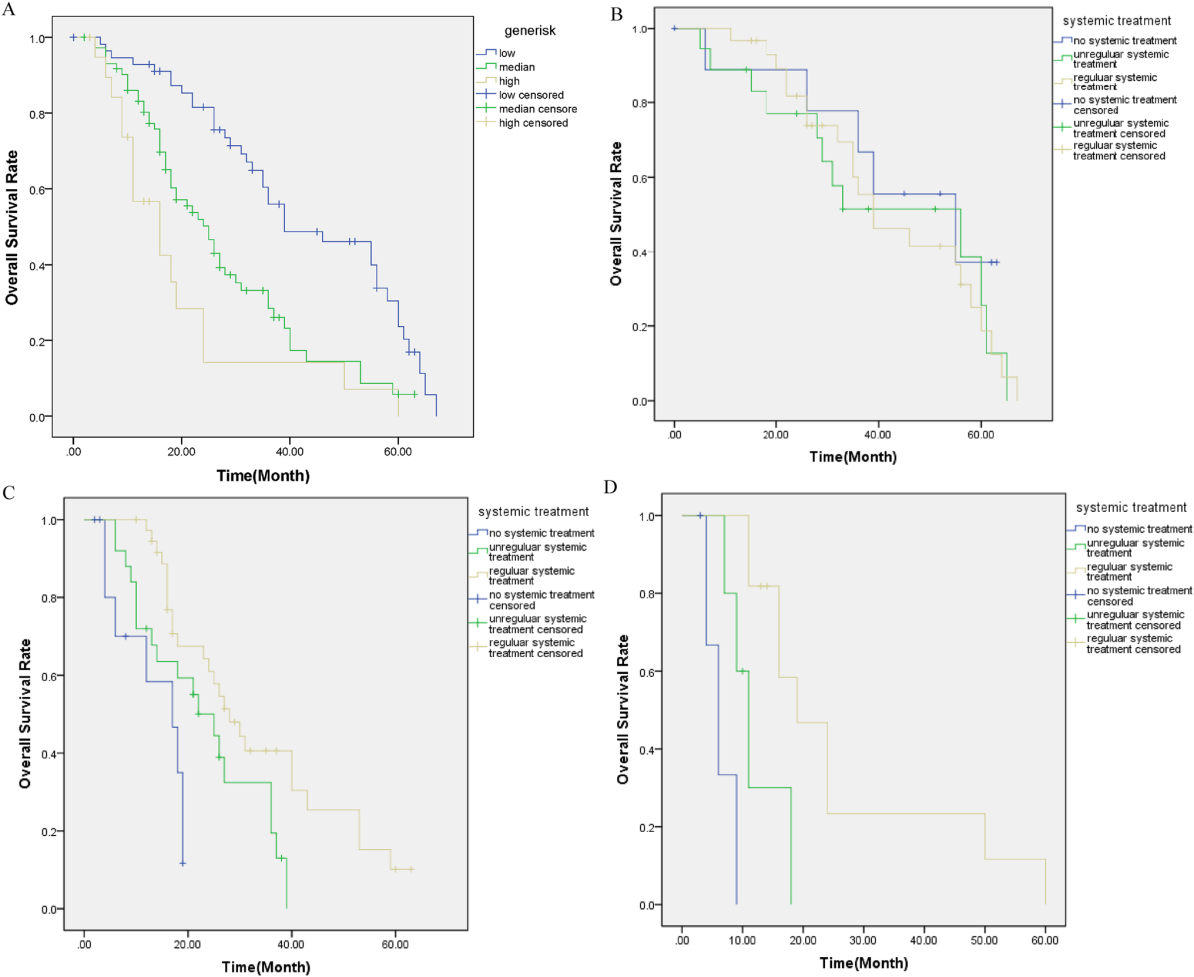
The overall survival under different gene risk. **A** The survival cure of CCA at different gene risk. **B**–**D** The survival curve of CCA received different kinds of systematic treatment at low, median, and high gene risk condition, respectively

The genes whose mutation frequency higher than 5% in the training cohort were selected to be prognostic candidates of CCA. SMAD4, MUC16, BRCA2, KRAS, ERBB2, NF1, TERT, and MDM2 were found to be associated with OS of CCA in the univariable Cox analysis, in those MUC16, ERBB2, and MDM2 did not show significant association with OS in the multivariable Cox analysis. Therefore, SMAD4, BRCA2, KRAS, NF1, and TERT formed the gene mutation score, which divided CCA patients into three groups. The detailed information is shown in Fig. 2. The OS of the low-risk group was 42.7 ± 2.7 ms (95% CI 37.5–48.0), the median-risk group was 27.5 ± 2.1 ms (95% CI 23.3–31.7), and the high-risk group was 19.8 ± 4.0 ms (95% CI 11.8–27.8) (*p* < 0.001), respectively. The survival curve is shown in Fig. 3a.

### The gene risk can be used as a prediction of concurrent systemic chemotherapy

Considering many gene mutation was associated with OS of CCA in our study, and different treatment response revealed in patients, we analyzed the relationship with the gene mutation score and systemic chemotherapy, and interestingly found that the gene mutation score can be used as a prediction of concurrent systemic chemotherapy. The systemic chemotherapy condition of patients was divided into three kinds: no systemic treatment, unregular systemic treatment, and regular systemic treatment. The regular systemic treatment (RST) means the patient received full course chemotherapy and adjusted timely according to multi-disciplinary team suggestion. No systemic treatment (NST) means the patient received no chemotherapy for whatever reason. The unregular systemic treatment (UST) was the median condition.

The OS of NST, UST, and RST patients in the low-risk group were 45.4 ± 6.4 ms (95% CI 32.9–52.9), 41.5 ± 5.4 ms (95% CI 30.9–52.0), and 42.7 ± 3.5 ms (95% CI 35.9–49.5), (*p* > 0.05), respectively. In the median-risk group, the OS in those three groups were 13.5 ± 2.1 ms (95% CI 9.4–17.7), 23.0 ± 2.5 ms (95% CI 18.0–27.9), and 33.3 ± 3.1 ms (95% CI 27.2–39.4), (*p* < 0.05), respectively. In the high-risk group, the OS were 6.3 ± 1.5 ms (95% CI 3.5–9.2), 11.9 ± 2.4 ms (95% CI 7.2–16.6), and 26.4 ± 5.7 ms (95% CI 14.9–37.9), (*p* < 0.05), respectively. Systemic treatment improved the OS of CCA in the median- and high-risk groups (Fig. 3b, c), but not in the low-risk group (Fig. 3d).

### Independent prognostic risk factors selection

**Fig. 4:**
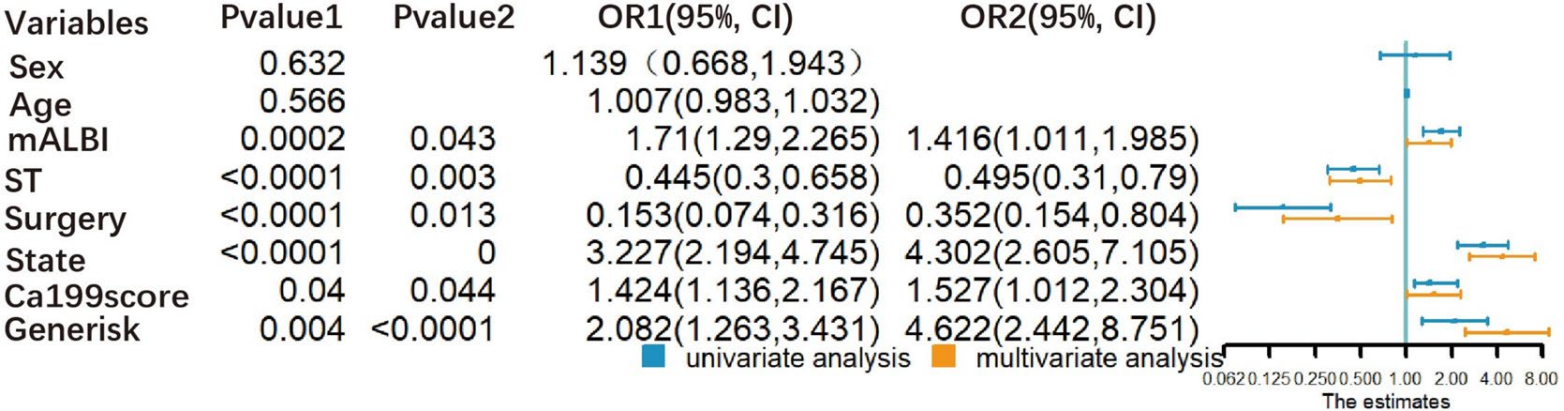
The relationship of clinicopathological information, gene risk and overall survival of CCA. Note: Pvalue1: univariable Cox analysis. Pvalue2: multivariable Cox analysis. *OR* Odds ratio

All clinicopathological information and the gene risk were analyzed if associated with the OS of CCA. Items both were positively related with OS in the univariable and multivariable Cox analyses were selected to be the independent prognostic risk factors. The surgery, stage, systemic treatment, mALBI, Ca19-9 Score, and gene risk were on the official list. Detailed information is shown in Fig. 4.

### The nomogram including the gene risk predicted the prognosis of CCA better than not

**Fig. 5:**
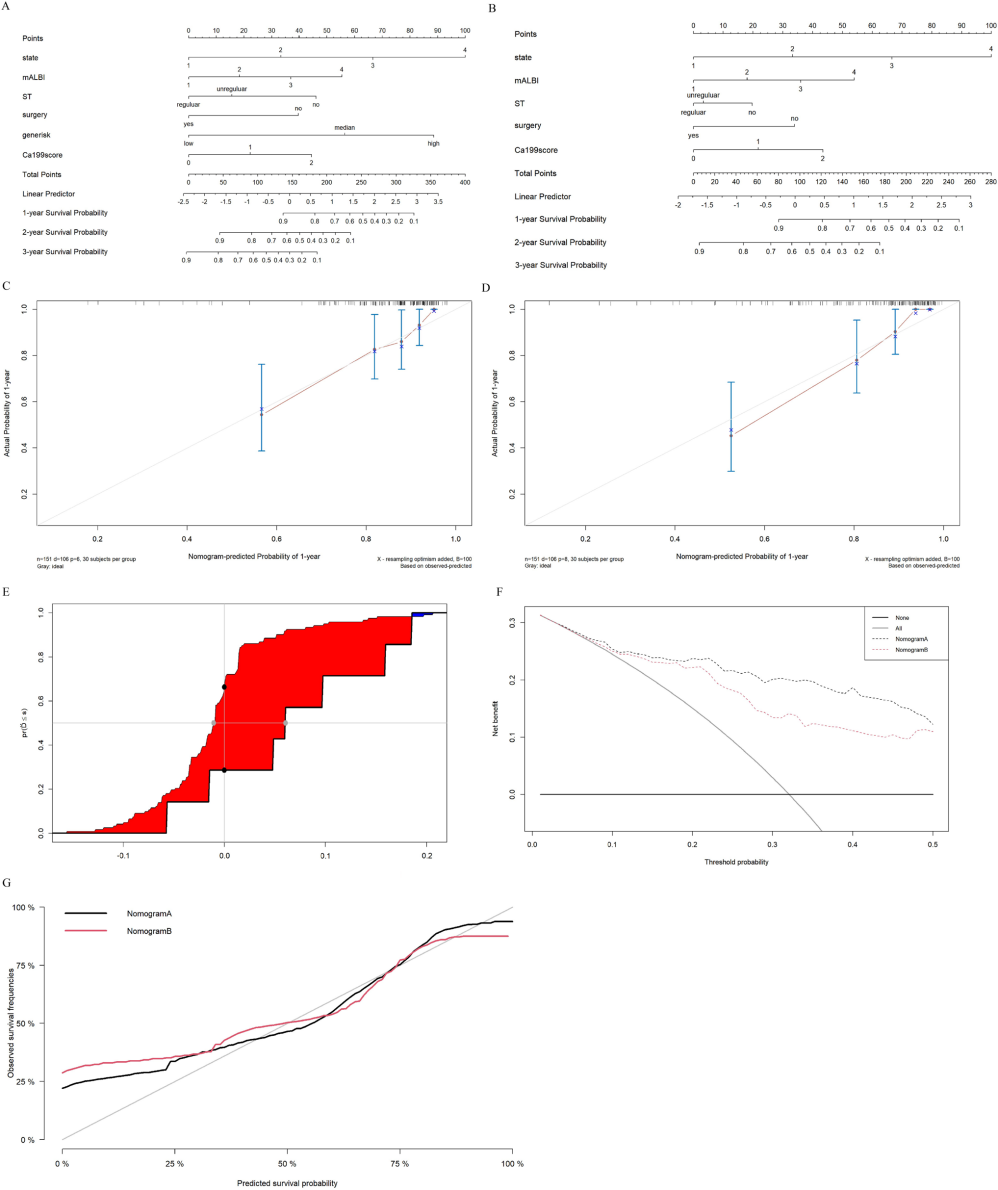
The comparison of Nomograms with or without gene risk and the validation information. **A**, **B** Nomogram with and without gene risk; **C**, **D** the calibration curve of Nomogram A and B; **E** the integrated discrimination improvement (IDI) curve of nomogram A, B; **F** the decision curve analysis (DCA) of Nomogram A and B; **G** the calibration plot in the validation cohort

Nomograms were generated using multivariate analysis to predict OS in the training cohort (Fig. 5A, B). The predictors in nomogram A included surgery, stage, systemic treatment, mALBI, Ca19-9 Score, and gene risk. Meanwhile, the Nomogram B included all the clinicopathological information but excluded the gene risk to evaluate its influence in the OS prediction of CCA. The C-indexes of nomogram A and B were 0.779 (95% CI 0.693–0.865) and 0.725 (95% CI 0.619–0.831), (*p* < 0.01), respectively. The calibration curve of Nomogram A was closer with the ideal status than Nomogram B (Fig. 5C, D).The integrated discrimination improvement (IDI) of nomogram A to B was 0.079 (Fig. 5E). When the ratio is between 0.1 and 0.5, the net benefit ratio of Nomogram A was higher than Nomogram B (Fig. 5F). The calibration plot in the validation cohort confirmed the good prediction of the nomograms (Fig. 5G).

## Discussion

CCA is a cholangiocyte original malignancy within the biliary tree with poor prognosis, and is divided into three categories according to anatomical location as intrahepatic (iCCA), perihilar (pCCA), and distal (dCCA) with different strategies for clinical management and prognosis. Surgical treatment and liver transplantation were the first choice if available. For metastatic CCA, systemic treatment including gemcitabine and cisplatin or EGFR inhibitors was recommended. Despite great efforts have been attempted, the prognosis of CCA is not satisfactorily improved. Apart from the CCA grade, many factors were reported to be associated with CCA prognosis, such as hepatic viral infection history, the baseline liver function and Ca19-9 level, radical resection or not, and systemic treatment status. Recently, according to the cost reduction and popularizing of gene testing technical, more and more gene mutations were reported to be related with the prognosis of CCA. To better estimate the prognosis of CCA, we selected the prognosis-related clinical factors and gene mutations, and build nomograms including the gene risk score or not, compared to their accuracy and validation through the external test cohort.

Many gene mutations might occur in CCA progression. In this study, the big panel of 450 genes test showed the mutation information was similar between the training and validation cohort, and also similar to the studies reported before (Montal et al. 2020; Yoon et al. 2021). TP53, KRAS, SMAD4, and CDKN2A were the TOP4 frequency mutations both in the training and validation cohorts. SMAD4, BRCA2, KRAS, NF1, and TERT were selected to form the gene mutation score after univariable and multivariable Cox analyses. SMAD4 is reported as an independent prognostic biomarker of CCA. SMAD4 suppresses CCA proliferation, migration, invasion, and sensitivity to Pemigatinib by regulating the phosphorylation and intracellular localization of β-catenin (Liu et al. 2022). BRCA2-related CCA was uncommon. If BRCA2 is positive, Olaparib and Pembrolizumab might be helpful for advanced CCA (Zhou et al. 2022; Li et al. 2021; Costa et al. 2023). In addition to its own anti-tumor effects, Olaparib sensitizes cholangiocarcinoma cells to radiation if BRCA2 is positive (Mao et al. 2018). KRAS mutation was prevalent, and can be considered with targeted therapies (Nguyen et al. 2021). The molecular characterization of CCA could predict chemotherapy and Programmed Death 1/Programmed Death-Ligand 1 blockade responses (Yoon et al. 2021). Interestingly, we also found that the gene mutation score of CCA was associated with the response of systematic treatment (ST) in our study. In low-risk group, patients seemed not get beneficial outcome from ST, exhibiting potentiality of selecting more profitable patients to receive ST according to the gene mutation score, which might decrease the unnecessary overtreatment and improve the patients’ quality of life.

Ca19-9 was a traditional biomarker of CCA for diagnosis and prognosis (Liang et al. 2015). The preoperative CA19-9 level was associated with clinicopathological factors and overall survival. Persistent high CA19-9 level is after resection of CCA and R1 section, especially in the preoperative high-level group. To better reveal the influence of preoperative CA19-9 level and postoperative improvement of CCA prognosis, we proposed a new index named ‘CA19-9 Score’ considering both pre- and postoperative level. In our study, CA19-9 Score was significantly gradient related with OS of CCA. The high level at preoperative stage mostly related with high tumor load or lymphatic metastasis, and high level at postoperative stage mostly related with residual tumor, both were independent risk factors for OS (Lee et al. 2021).

Child–Pugh (CP) grade has been widely used to evaluate liver function and postoperative outcomes in biliary tract malignancy. (Wang et al. 2018) reported a novel alternative model of liver function, called albumin–bilirubin (ALBI) grade in 2018, showed a better prediction in extrahepatic cholangiocarcinoma. Tokunaga T. et al. modified this model by dividing the grade 2 into grade 2a and 2b, exhibited eligible prediction for second-line therapies of hepatocarcinoma (Tokunaga et al. 2021). In this study, we found that the OS of patients with middle grade liver function also quite different. For convenience to analysis, we divided the liver function by a new modified ALBI grade of grade 1, 2, 3, and 4. The lower grade usually means better nutrition and fewer hepatocellular damage, mostly stands for better prognosis. The results of Cox analysis and nomogram confirmed this.

Surgical treatment for extrahepatic CCA included pancreaticoduodenectomy (PD), bile duct segmental resection (BDR), and liver transplantation. BDR was comparable in prognosis to PD in middle bile duct cancer, with a 5-year survival rate 44% for BDR and 51% for PD (*p* = 0.72) (Akita et al. 2020). For patients with poor general condition, BDR was recommended as a less invasive and lower morbidity technique. Both PD and BDR of patients have better overall survival than who did not undergo curative resection and palliative chemotherapy (Saragih et al. 2022), which was verified in our study. After curative resection, microscopic residual tumor was respected as a high-risk factor of recurrence and death, and adjuvant concurrent chemo-radiation therapy could reduce this risk significantly (Lee et al. 2018). Complete surgical resection did not mean cure, the 5-yr survival can be as low as 11% (Anderson and Kim 2009). Adjuvant therapy (AT) has the potential to play a crucial role in prolonging survival and local control, especially in patients with node positive disease (Krasnick et al. 2018). In our study, systematic treatment also included epidermal growth factor receptor (EGFR) and programmed death-1(PD-1) inhibitors. Patients with systematic treatment have lower probability of recurrence and long survival. Similar to the previous study, we also found that tumor location of extrahepatic CCA does not independently predict cancer-specific survival after resection (Gaag et al. 2012).

In this study, we developed two nomograms using the clinical pathological and treatment information with or without the gene mutation score. The Nomogram with gene risk showed a better accuracy and confirmed by the validation cohort. Along with the progress and lower cost of next-generation sequencing (NGS), gene mutation test might be a routine check after CCA diagnosed, which can better guide clinicians choosing target therapeutics, decide ST or not, and predict the prognosis.

This study has limitations. Despite risk factors which have been included in this study, systemic immune inflammation index (SII) was reported as an independent risk factor of postoperative OS following curative-intent resection of eCCA with controversy (Toyoda et al. 2022; Sahara et al. 2021; Reames and Rocha 2021). C-reactive protein-to-albumin ratio (CAR) was another valuable prognostic score in patients with resected extrahepatic cholangiocarcinoma (Asakura et al. 2022). Lacking of the relevant data, those indexes were not included in this study. Another limitation is the small sample size of the validation cohort and data were from the same centers of the training cohort in different stages, not absolutely independent external validation.

## Conclusion

The gene risk is a reliable prognostic tool for OS in patients with CCA and might be able to predict which patients benefit from concurrent chemotherapy. The nomogram combined with the clinicopathological and gene risk showed a better accuracy in predicting the prognosis of CCA. It has the potential to guide treatment decisions for patients at different risks and predicts the prognosis of CCA patients.

## Supplementary Information

Below is the link to the electronic supplementary material.

Supplementary file1 (XLSX 76 KB)

## Data Availability

The data used to support the findings of this study are available from the corresponding author upon request.
